# News and Coming Events

**Published:** 1908-09-19

**Authors:** 


### News and Coming Events.

ROYAL NAVY MEDICAL SERVICE.

An examination of candidates for entry into the Medical Department of the Royal Navy will be held on November 2 and following days at Examination Hall, Victoria Embankment. Fifteen commissions will be offered for competition. All information as to conditions of service, rank, and pay, and also the forms to be filled up by candidates, will be supplied on application to the Medical Director General, Admiralty, 18 Victoria Street, London, S.W.

Mr. F. D. S. Jackson, M.R.C.S., L.R.C.P.Lond., has been appointed anæsthetist at the Evelina Hospital for Sick Children, Southwark, London, S.E.

We regret to announce the sudden death of Dr. Mary Hamilton (L.R.C.P. and S., Edinburgh), of Pengarth, St. Agnes, Cornwall, in Edinburgh on Monday, September 7. Her body was laid beside her father's, the late Sir Robert G. C. Hamilton, in Richmond Cemetery, Surrey, on Saturday, September 14.

A special souvenir programme is being arranged for sale at the morning performance, to be held at the end of October, at the St. James's Theatre, on behalf of the Royal National Orthopædic Hospital. Among those who have already promised contributions to the souvenir are Mrs. W. K. Clifford, Miss Marie Corelli, Mr. C. J. Cutcliffe-Hyne, Mr. Bernard Partridge, Mr. Lewis Baumer, Mr. John Hassall, and Sir A. C. Mackenzie. The hospital committee hopes to announce the names of further contributors in the course of a few days.

Preparations have now been completed for the new Commission under Colonel Sir David Bruce, which, as previously announced, is being despatched to East Africa by the Imperial Government under the direction of the Royal Society to investigate Sleeping Sickness. Within the next few days the members will leave on board the German steamship *Admiral*, reaching Uganda about the middle of October. On arriving at Mombasa the Commission will travel by the Uganda Railway to the terminus at Port Florence, whence the lake will be crossed to Kampala. The headquarters of the work will be selected two miles from the lake shore in a wild and depopulated region in the Province of Chagwe, where the Uganda Government has been preparing a laboratory and station for the purposes of the mission.

Fleet-Surgeon Richard Eustace, who died at Bournemouth on September 10, aged 75, qualified in 1853 and joined the Royal Navy in the following year, serving in the Baltic during the war with Russia. He was present at the temporary occupation of Petropaulovski in May, 1855, and at the close of the war he received the Baltic medal. In 1862 he was promoted Staff-Surgeon, and served on the Gold Coast during the Ashantee War, 1873-74. He received the thanks of the Admiralty for his care of the sick on board the troopship, the Ashantee medal, and special promotion in March, 1874, to Fleet-Surgeon. He was awarded the Sir Gilbert Blane gold medal for his medical and surgical journal of 1873. The degree of M.D. *Honoris Causa* was conferred upon him by the Queen's University in Ireland in 1876. Dr. Eustace retired from active service in the Navy in 1874.

The Dublin *Gazette* announces that the Lord Lieutenant has been pleased to appoint Mr. Ambrose A. Kelly and Mr. Joseph F. O'Carroll, M.D., to be members of the Board of Superintendence of Dublin Hospitals.

Mr. Rudyard Kipling will distribute the prizes at the opening of the winter session of the Middlesex Hospital Medical School on Thursday, October 1, after an introductory address by Dr. A. M. Kellas.

Dr. Herbert Jones, the present medical officer of health for the Herefordshire combined districts and Bromyard Urban District, has been appointed by the Herefordshire County Council county medical officer of health.

The authorities of the London Hospital have received intimation from Dr. Tyrnauer, of Carlsbad, inventor of the hot-air appliances in use at the baths of that health resort, that Princess Hatzfeldt has expressed her intention to present a complete outfit of the apparatus for the use of the London Hospital. It is already in course of construction at Wiesbaden, and, it is hoped, will be in position at the hospital during October.

At the opening of the winter session at St. George's Hospital Medical School on October 1, 1908, the introductory address will be given in the large theatre at 4 p.m. by Dr. Charles Slater on "The Laboratory in Medical Education and Practice." The annual dinner will take place at the Whitehall Rooms of the Hôtel Metropole, at 6.30 for 7 p.m., under the chairmanship of Dr. T. T. Whipham. Tickets for the dinner, price £1 1s., may be obtained from the Hon. Secretaries of the Hospital Dinner, St. George's Hospital, S.W.

We much regret to announce the death, on September 10, of Mr. Edward Percy Paton, M.D., M.S., F.R.C.S., Assistant Surgeon to the Westminster Hospital and Dean of the Medical School, at the age of forty-one, after a long illness in the Westminster Hospital. Mr. Paton was educated at St. Bartholomew's Hospital, and qualified in 1889. His record for the M.B. and B.S. examinations of the University of London was brilliant, for he obtained the University scholarships and gold medals both in surgery and in obstetric medicine, as well as honours in medicine, forensic medicine, and physiology. In 1891 he became M.D., in 1892 F.R.C.S. Eng., and in 1894 M.S. London. After serving as house-surgeon and assistant demonstrator of anatomy at St. Bartholomew's, and surgical registrar and anæsthetist at the Children's Hospital, Great Ormond Street, Mr. Paton became successively surgical registrar, demonstrator of anatomy, lecturer on pathology and surgeon to out-patients at the Westminster Hospital, with which institution he thenceforward became intimately associated. Not only were his academic attainments of a high order, but he was a surgeon of great practical ability, a good and popular teacher, and (as his tenure of office as Dean of the Westminster Hospital Medical School showed) a sound man of business. His early death cuts off a brilliant career, and he will be keenly regretted, personally and professionally, by everyone connected with the Westminster Hospital, and by a wide circle of other friends. His surgical opinion was highly esteemed, and he was a finished and dexterous operator. His death, coming so soon after that of his colleague, Dr. Bertram Abrahams, is a severe loss to the Westminster Hospital and its school.

### LARGE BEQUESTS TO CHARITIES.

Mrs. Jane Churton, of Watergate Flags, Chester, bequeathed £2,400 to the Chester General Infirmary to establish, endow, and maintain two beds in remembrance of her father and mother, John and Henrietta Myers, and her two sisters Anne and Elizabeth Myers; £2,000 to the Chester General Infirmary for general purposes, and £2,000 to the Royal Albert Asylum, Lancaster; and £1,000 to the Children's Convalescent Home, West Kirby, Cheshire.

Mrs. Mary Bridget Johnston, of the Manor House, Widcombe, near Bath, Somerset, bequeathed £2,000 each to the Cumberland Benevolent Institution, London, the Society for the Relief of Distress, 78 Jermyn Street, London, and £1,000 each to the British Home for Incurables, London, the Royal Free Hospital, Gray's Inn Road, and the Cancer Hospital, Fulham Road, London.

Miss Charlotte Frances Foster, of Norwich, bequeathed £200 to the Norfolk and Norwich Eye Infirmary, £500 to the Jenny Lind Infirmary for Sick Children, and £1,000 to the Norfolk and Norwich Hospital.

Mr. George Cooper, of Ashton-on-Mersey, Chester, who died on August 7 last at Brighton, bequeathed £1,000 to the Salford Royal Hospital.

---

The President (the Right Honourable the Lord Mayor) and Board of the Royal Waterloo Hospital, S.E., wish to express their thanks to an anonymous donor of £1,000 to the funds of this most deserving institution. One ward is still unopened owing to insufficiency of annual income, but it is hoped that this may shortly be remedied, and the fullest use made of the institution.

---

Brigade-Surgeon Robert Lidderdale, M.D., who died at Torquay on Sept. 9, aged seventy-three years, served for thirty years in the Army in India, after qualifying at Edinburgh in 1857. He served in the Mutiny and with the Punjab frontier force. For these services in the field he received a medal and clasp. His chief medical work was in connection with the Vaccination Department for Bengal. Dr. Lidderdale was placed on the Retired List in March 1885. He was well known as an entomologist, and when attached to a regiment serving in Bhutan he discovered an extremely rare butterfly which bears his name. His collection of day-flying moths was unique, and was purchased some years ago by the British Museum.

---

A Reuter's telegram from St. Petersburg, dated September 15, announces that the towns of St. Petersburg, Kieff, Ekaterinoslav, and Tashkent, the Governments of St. Petersburg, Moscow, Tchernigoff, and Tomsk, the Transcaspian territory, the Provinces of Syr-Daria, Samarkand, and Ferghana, and the railways in the St. Petersburg district have been officially declared to be infected with cholera or in danger of infection. From August 10 to September 14 the number of cases in St. Petersburg was 401, of which 98 were fatal. Since the outbreak of the epidemic there have been in the whole of Russia 6,747 cases, 3,130 of which resulted in death. The regulations of the Local Government Board dealing with suspected ships are being strictly enforced in the Port of London and at the other ports in the United Kingdom. All vessels coming from infected Russian ports are being stopped at Gravesend, where there is a close medical examination. At Liverpool the port sanitary authority has decided that any ship coming from an infected port shall be detained in quarantine for a period during which symptoms would disclose themselves.

The Hon. Secretaries of King Edward's Hospital Fund for London have received at the Bank of England, from the executors of the late Mr. Samuel Lewis, the sum of £2,500, being the ninth payment on account of the legacy of £250,000 bequeathed by him to the fund.

---

His Excellency Professor von Leyden, of Berlin, and his Excellency Professor Czerny, of Heidelberg, have been elected Presidents, and Professors Pierre Marie, of Paris, and Fibiger, of Copenhagen, Vice-Presidents, of the International Association for the Investigation of Cancer, founded at Berlin on May 23 of this year.

---

Under the will of Mr. Paul Hirsch, of Alma House, Headingley, Leeds, woollen merchant, the following sums are left to medical charities: £500 to the Hospital for Women and Children at Leeds; £200 to the Leeds Infirmary; £200 to the Leeds Dispensary; £200 to the Leeds Association for Prevention and Cure of Tuberculosis; £200 to the Leeds Maternity Home.

Lieut.-Col. (local Colonel) George Douglas Hunter, D.S.O., Royal Army Medical Corps, attached to the Egyptian Army, has received his Majesty's permission to accept the Second Class of the Imperial Ottoman Order of the Medjidieh; and Major George Dansey-Browning, Royal Army Medical Corps, the Fourth Class of the Order of the Osmanieh, conferred upon them by the Khedive of Egypt in recognition of valuable services rendered by them.

Messrs. T. C. and E. C. Jack, of Edinburgh, and Henrietta Street, London, have purchased the well-known reference-book entitled "Parnell's Reference Book," and announce its re-issue in the autumn under the title of "Jack's Reference Book." The book consists of over 1,000 pages of small type, and the publishers have arranged to keep the type standing so that alterations may be made as they become necessary. The book will therefore be continually kept up to date, and in point of extent will certainly be the cheapest and handiest encyclopædia now for sale in Britain.

